# Spatiotemporal Distribution of Phytoplankton Functional Groups in Baihua Reservoir: Implications for Ecosystem Management

**DOI:** 10.3390/biology14040333

**Published:** 2025-03-25

**Authors:** Zhongxiu Yuan, Yan Chen, Si Zhou, Yugui Peng, Jing Xiao, Qiuhua Li

**Affiliations:** 1 Key Laboratory for Information System of Mountainous Area and Protection of Ecological Environment of Guizhou Province, Guizhou Normal University, Guiyang 550001, China; 18708659007@163.com (Z.Y.); 2321006529@gznu.edu.cn (Y.P.); jing-xiao2022@gznu.edu.cn (J.X.); 2 Guizhou Key Laboratory of Advanced Computing, Guizhou Normal University, Guiyang 550001, China; cy@gznu.edu.cn; 3 Guizhou International Cooperative Research Base-International Joint Research Center for Water Ecology, Guiyang 550001, China; 4 Guizhou Province Field Scientific Observation and Research Station of Hongfeng Lake Reservoir Ecosystem, Guiyang 551499, China; 5 School of Cyber Science and Technology, Guizhou Normal University, Guiyang 550001, China; 6 Department of Aquatic Ecological and Environmental Research, Guizhou Provincial Environmental Science Research and Design Institute, Guiyang 550002, China; zhousi2022@163.com

**Keywords:** phytoplankton, functional groups, spatiotemporal succession, community stability, Baihua Reservoir

## Abstract

Functional groups are an effective method for assessing water quality. From January 2020 to December 2023, the phytoplankton and environmental variables at five sites in Baihua Reservoir (BHR) were collected once a month. The succession rate (SR) and the average variation degree (AVD) of the functional groups were determined, and the corresponding driving factors were analyzed by using the Random Forest model, hierarchical partitioning, and Mantel test. A total of 95 phytoplankton species belonging to 7 taxonomic categories were identified, which can be divided into 27 functional groups and 8 dominant functional groups (B, D, L_0_, P, S1, W1, W2, Y). B, L_0_, and Y occupied dominant positions in spatiotemporal succession, indicating that the water body was in a mesotrophic to eutrophication state. Water temperature, total nitrogen, and transparency were the key factors driving the functional groups’ succession. Total nitrogen, total phosphorus, permanganate index, and dissolved oxygen were significantly positively correlated with AVD (*n* = 230; *p* < 0.01). SR not only directly positively affected AVD (*n* = 230; *p* < 0.05) but also indirectly affected AVD by affecting physicochemical factors. Understanding the relationship between the succession, stability, and environmental factors of functional groups is of great significance for algae management and the prevention of water bloom.

## Simple Summary

Phytoplankton are typically the most significant primary producer in aquatic ecosystems, and the classification of phytoplankton into functional groups is an effective method for assessing water quality. In this paper, the succession and community stability of functional groups were explored in Baihua Reservoir. The study demonstrated that the water body of Baihua Reservoir was in a mesotrophic to eutrophic state. The functional group succession was most influenced by water temperature, total nitrogen, and transparency. The driving factors of stability were total nitrogen, total phosphorus, permanganate index, dissolved oxygen, and pH, and amongst them, the permanganate index had the most significant effect on the average variation degree index. There was a significant positive correlation between succession rate and average variation degree index. In order to control the outbreak of algal blooms in Baihua Reservoir, the nutrient concentration and Bacillariophyta abundance in the reservoir should be controlled. Algal management, the health of aquatic ecosystems, and an understanding of physical/chemical relationships are crucial for managing algal biomass leading to blooms in the reservoir.

## 1. Introduction

Phytoplankton serve as the dominant primary producers within most aquatic ecosystems and integral to the material cycling and energy transfer processes of ecosystems [1]. Phytoplankton possess a short life cycle, and their community structure can rapidly reflect alterations in the physical/chemical aquatic environment, revealing the state of water eutrophication [2]. However, the traditional species classification method based solely on morphological structure may deviate from actual habitat characteristics and conditions. Therefore, Reynolds proposed the idea of functional groups, aiming to illustrate the relationship between phytoplankton communities and environmental characteristics [3]. Functional groups allow phytoplankton with similar or identical physiological, morphological, and ecological characteristics to be categorized. Compared to traditional classification methods, functional groups offer a more effective way to evaluate habitat characteristics by combining species with similar traits [4].

Functional groups streamline traditional taxonomic systems by grouping organisms based on ecological roles, enabling predictions about dominance under specific environmental conditions. The dominance of particular functional groups can further serve as indicators of habitat-specific ecological disturbances, such as eutrophication or pollution.

At present, a total of 38 groups of phytoplankton functional groups and their identification characteristics and representative species have been determined [5]. By classifying phytoplankton into functional groups, this study reveals how functional traits shape community dynamics and identifies key environmental drivers in the study area.

Succession is a process of change driven by disparities in community growth rates, resulting in alterations to the biomass of each species. Due to the rapid growth cycle of phytoplankton, the succession rate (SR) can be employed to examine the transformation process of phytoplankton [6]. The SR can represent the gradual and abrupt variations in phytoplankton, and the SR based on the biomass of phytoplankton functional groups can better reflect the composition of community structure and environmental ecological characteristics [7], so long as patchiness of species is not confounding. Community stability includes resistance stability and resilience stability, which refer to the ability to maintain the combination of species and the quantitative relationship of species in a certain period of time, and the ability to restore to the original equilibrium state in the case of disturbance [8]. A stable community can provide sustainable ecosystem services, such as carbon fixation, oxygen production, water quality maintenance, and biodiversity maintenance. If the community is unstable, it may lead to the decline of ecosystem services and even ecosystem collapse [9]. The stability of phytoplankton can effectively characterize the change in phytoplankton community structure and ecosystem function [10]. Phytoplankton serve as the main producer within aquatic ecosystems, and the succession and stability of their functional groups are directly linked to the health status and service function of an ecosystem.

Southwest China is a typical karst landscape with many karst reservoirs, which are important for regional water supply and biodiversity maintenance. Numerous studies have been conducted to examine water quality and habitat changes in lakes, reservoirs, and rivers from a functional group perspective [11,12,13,14]. However, research on the functional groups has not been done in Baihua Reservoir (BHR), Southwest China.

This study aims to investigate the spatiotemporal dynamics of phytoplankton communities and their functional groups in BHR, with a focus on the following three interrelated objectives: to quantify the succession patterns of functional groups across temporal and spatial gradients and evaluate their coupling mechanisms with key water quality parameters; to identify the stability of functional groups assemblages and elucidate their driving factors through environmental and biotic interactions; to understand the relationship between functional group succession and community stability in BHR, and reveal the direct and indirect effects between them. This information has a guiding role for the regulation and scientific management of algae in the reservoir, and is of great significance for the structure, function, health status, and prediction of future changes in aquatic ecosystem.

## 2. Materials and Methods

### 2.1. Study Area

Baihua Reservoir (26°35′–26°41′ N, 106°27′–106°32′ E), located in the Karst region of southwest China, is part of the Maotiao River basin within the Wujiang River system, a tributary of the Yangtze River. BHR features a maximum water depth of 45 m and an average water depth of 10.8 m. BHR has a basin area of 1895 km^2^, a total capacity of 1.82 × 10^8^ m^3^ and a total annual water supply of 28.72 × 10^6^ m^3^, it serves as a crucial supply of drinking water for Guiyang City and is vital for electricity generation, flood management, and agricultural irrigation, as well as tourism. Its water quality is intricately linked to both the aquatic ecological environment and the living standards of the local inhabitants [15].

**Figure 1:**
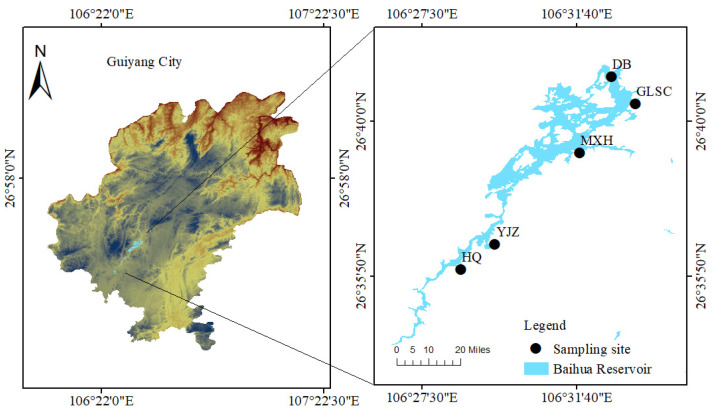
The location of Baihua Reservoir (BHR) and the position of the sampling sites.

BHR is an elongated reservoir and a typical karst reservoir in Guizhou Province, characterized by numerous rural settlements and agritourism facilities along its shores. Sampling sites were strategically established along the water flow direction, including the inlet, shallow-water zone, deep-water zone, water intake, and outlet. Based on the morphological characteristics, geographic conditions, and hydrological conditions of BHR, this study established five sampling sites along the reservoir from its head to its tail waters, namely Huaqiao (HQ), Yanjiaozhai (YJZ), Maixihe (MXH), Guilvshuichang (GLSC), and Daba (DB) (Figure 1). The HQ segment exhibits dense residential settlements with intensive human activities and substantial domestic sewage discharge. However, its limited water surface area constrains the self-purification capacity of the aquatic system. In contrast, the MXH experiences moderate anthropogenic pressure compared to HQ. Although the YJZ segment has fewer residential areas, it faces compounded challenges: receiving inflow from the upstream HQ segment and suffering from suboptimal operation of small-scale domestic wastewater treatment plants within its watershed, resulting in the direct discharge of domestic sewage, aquaculture wastewater, and agricultural runoff into the reservoir.

### 2.2. Samples Collection and Analysis

Monthly collections of water and phytoplankton samples occurred at five locations within BHR between January 2020 and December 2023. A portable multi-parameter water quality analyzer (HANNA, HI 98194, Shenzhen, China) was utilized for on-site measures of water temperature (WT), dissolved oxygen (DO), and pH, while transparency (SD) was assessed using a Secchi disk in situ.

A volume of 3 L of water samples were taken at each sampling point using a 5 L sampler and stored in 1.5 L plastic bottles made of polyethylene. A 1.5 L water sample was used for phytoplankton quantification, which was immediately fixed with a 1.5% Lugol’s solution after sampling. In the laboratory, the sample was allowed to settle for 24–48 h via sedimentation, after which the supernatant was removed using a siphon method to concentrate the sample to a final volume of 30 mL. Each concentrated sample was labeled with critical metadata, including collection time, location, pre-concentration volume, and post-concentration volume. For taxonomic identification and enumeration, a biological microscope (Olympus CX43, Shanghai, China) was employed, following the classification methodologies outlined in *Freshwater Algae of China: Systematics, Taxonomy, and Ecology*. Prior to counting, the concentrated sample was thoroughly homogenized by agitation. A 100 μL aliquot was pipetted into a 0.1 mL counting chamber (Sedgwick-Rafter cell), covered with a coverslip to eliminate air bubbles, and analyzed using the ocular grid method under 10 × 40 magnification. Phytoplankton abundance (cells/L) was calculated based on the counted individuals per taxon, standardized to the original sample volume. The other 1.5 L was used to measure the concentration of total nitrogen (TN), total phosphorus (TP), permanganate index (COD_Mn_), and ammonia nitrogen (NH_3_-N) using the Chinese national standard for water quality testing procedures [16].

### 2.3. Data Analysis

Phytoplankton biovolume is calculated according to cell volume and density [17].

Biovolume = density × volume × abundance × 10^−9^ (1)

where the density of phytoplankton is 1 g/cm^3^; the unit of volume is um^3^; the unit of abundance is cells/L; and the unit of biomass is mg/L.

The McNaughton dominance index (Y) was employed to denote the dominant phytoplankton species [18].

(2) Y=(Ni/N)·fi

where Ni is the abundance of species i; N represents the total abundance of all species; fi is the occurrence frequency of the species in each sampling site; the phytoplankton species with Y ≥ 0.02 are deemed the dominant species; when Y > 0.1, they are the absolute dominant species.

The functional group succession rate (SR) was calculated as follows:

(3) $${SR}_{ba}=\int_{i=1}^{n}\frac{{|f}_{ib}-f_{ia}|}{b-a}$$

where f_ib_ represents the relative biomass of functional group i at time b, f_ia_ denotes the relative biomass of functional group i at time a, and n is the count of species within the community at times a and b [19].

The stability of the phytoplankton community was assessed using the average variation degree (AVD) index. Generally, lower AVD values correspond to greater stability within the community. AVD was calculated as follows.

(4) $$AVD=\frac{\sum_{i=1}^{n}\left|a_{i}\right|}{k\times n}$$

(5) $$\left|a_{i}\right|=\frac{\left|x_{i}-\bar{x_{i}}\right|}{\delta_{i}}$$

where k is the number of samples, n represents the count of species. a_i_ represents the variability of species i, x_i_ is the abundance of species i in each sample. The mean and standard deviation of the abundance for species i across all samples are represented by $\bar{x_{i}}$ and $\delta_{i}$, respectively.

SPSS Statistics 26 was employed to conduct a one-way analysis of variance (ANOVA) for assessing the variations in physical and chemical parameters of water bodies across different years. If the test results showed that there were significant differences between groups (*p* < 0.05), the specific difference groups were further determined by LSD post hoc test.

Principal Component Analysis (PCA) and Permutational Multivariate Analysis of Variance were utilized to assess the similarity among sampling sites. The key functional groups influencing succession were identified using Random Forest. The contribution of each environmental factor to the explanation of functional group succession was quantified through hierarchical partitioning. The correlation between environmental factors and AVD was assessed using the Mantel test. The PLS-PM was employed to analyze the interaction and correlation among the SR, AVD, and environmental factors. All other images were plotted using OriginPro2024 (Northampton, MA 01060, USA).

## 3. Results

### 3.1. Variations in Environmental Factors

**Figure 2:**
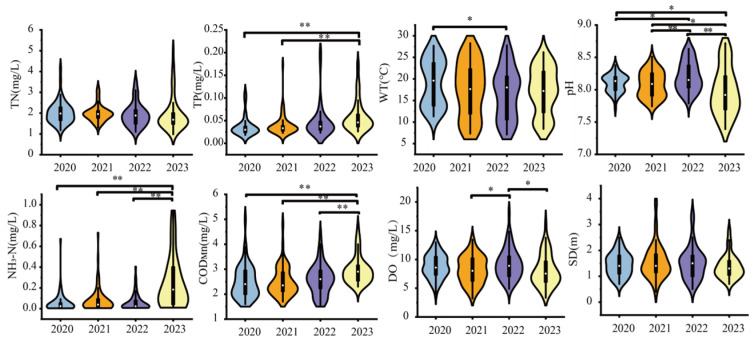
Changes in physicochemical indexes of BHR from 2020 to 2023. (TN, total nitrogen; TP, total phosphorus; NH_3_-N, ammonia nitrogen; COD_Mn_, permanganate index; DO, dissolved oxygen; SD, transparency); (the wider the width in the figure, the more data points in the data range; * denotes significant difference *p* < 0.05, ** denotes highly significant difference *p* < 0.01).

Environmental factors fluctuated greatly during the study period. Figure 2 illustrates the spatiotemporal variations in water environmental factors in BHR from 2020 to 2023. Temporal fluctuations were statistically significant (*n* = 230; *p* < 0.05) for all parameters except TN and SD. Chemical parameters were as follows: TN concentrations ranged from 0.98 to 5.00 mg/L (mean: 1.97 mg/L); TP varied between 0.02 and 0.20 mg/L (mean: 0.05 mg/L); NH_3_-N spanned 0.004–0.95 mg/L (mean: 0.12 mg/L); and COD_Mn_ fluctuated from 1.60 to 5.50 mg/L (mean: 2.64 mg/L). Physical parameters exhibited distinct patterns: WT ranged 7.10–28.30 °C (mean: 17.66 °C); pH maintained slight alkalinity (7.39–8.72; mean: 8.09); DO showed wide variability (3.20–18.10 mg/L; mean: 8.54 mg/L); and SD averaged 1.48 m (0.40–3.90 m). Notably, the HQ monitoring site consistently recorded maximum values for TN (5.00 mg/L), TP (0.20 mg/L), NH_3_-N (0.95 mg/L), COD_Mn_ (5.50 mg/L), WT (28.30 °C), pH (8.72), and DO (18.10 mg/L), alongside minimal SD (0.40 m), suggesting localized anthropogenic or hydrodynamic influences. Interannual analysis has shown that parameter levels were consistently lower in 2020 compared to the averages of 2021 and 2022. For example, TN levels decreased by 18% and COD_Mn_ levels decreased by 24%. These reductions may be attributed to the decrease in human activity during the COVID-19 restrictions.

### 3.2. Phytoplankton Community Structure

**Figure 3:**
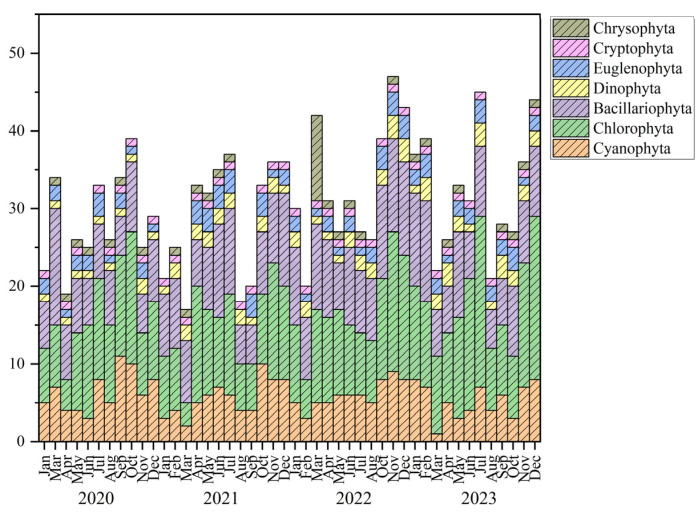
Composition of the phytoplankton community structure in BHR.

During the study period, the surface water of BHR was found to contain a total of 95 phytoplankton species belonging to 7 taxonomic categories. The Chlorophyta dominated the phytoplankton community with 43 species, constituting 45.26% of the total species count. This was followed by Bacillariophyta with 25 species, making up 26.32%, and Cyanophyta with 17 species, comprising 17.89%. Together, these three phyla accounted for 89.47% of the total phytoplankton species. There were fewer species in the phyla of Dinophyta, Euglenophyta, Cryptophyta, and Chrysophyta, which accounted for about 10.53%. The four-year study period, in descending order of the number of species, was 2023 (77 species), 2022 (67 species), 2020 (66 species), and 2021 (64 species) (Figure 3).

**Figure 4:**
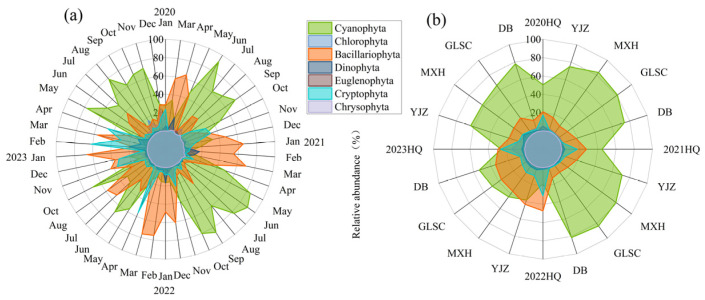
Temporal (**a**) and spatial (**b**) dynamics of phytoplankton relative abundance.

Figure 4 depicted the changes in phytoplankton relative abundance over time and space in BHR. In general, Cyanophyta constituted a significant portion, with Bacillariophyta being the next most prevalent. The phytoplankton abundance varied between 0.11 and 3.57 × 10^7^ cells/L. The average abundance of phytoplankton in 2020, 2021, 2022, and 2023 was 3.65 × 10^6^ cells/L, 4.85 × 10^6^ cells/L, 4.11 × 10^6^ cells/L, and 1.35 × 10^7^ cells/L, respectively. The average abundance of phytoplankton at HQ, YJZ, MXH, GLSC, and DB was 1.56 × 10^7^ cells/L, 1.37 × 10^7^ cells/L, 1.65 × 10^7^ cells/L, 1.56 × 10^7^ cells/L and 1.49 × 10^7^ cells/L, respectively.

From 2020 to 2023, the dominant phytoplankton community within the BHR ecosystem contained 26 species, which were distributed among seven divisions. The composition included seven species affiliated with Cyanophyta, seven in Bacillariophyta, five in Chlorophyta, four in Euglenophyta, one Dinophyta, one Chrysophyta, and one Cryptophyta phyla. Among these species, *Pseudanabaena limnetica*, *Synedra* sp., *Cyclotella* sp., *Achnanthes* sp., *Melosira* sp., *Peridinium* sp., and *Cryptomonas* sp. were the dominant species over the four years, and *Pseudanabaena limnetica* was the absolute dominant species during the study period. The dominant species show strong adaptability to the current environmental conditions and can effectively indicate the ecological environment.

**Figure 5:**
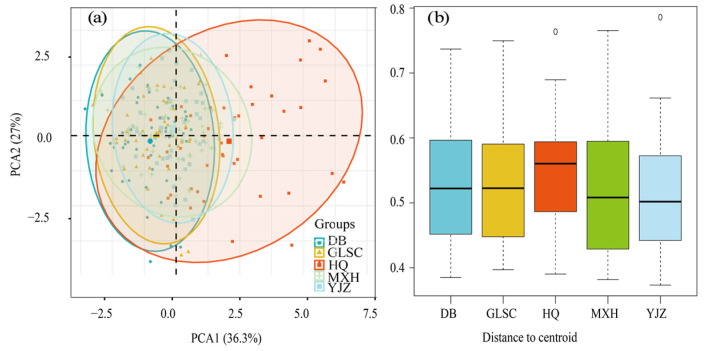
PCA (**a**) and displacement multivariate dispersion analysis (**b**) at different points of BHR.

Principal component analysis (PCA) was used to visualize the phytoplankton abundance and physicochemical data of the five BHR sites, and it was found that there was overlap between different sites. The permutation multivariate dispersion analysis was used to determine whether there were significant statistical differences between different sample groups [20]. The proportion of variance explained by each principal component in PCA is calculated by dividing the eigenvalue of that principal component by the total sum of the eigenvalues of all principal components. The first principal component accounts for 32.00% of the variance, while the second principal component contributes 27.50% to the variance. Except for the HQ site, the other four sites have a large overlap area, and the community similarity is high. According to the displacement multivariate dispersion analysis, there is spatial heterogeneity among the points of BHR (Figure 5). Hence, this research will explore the succession features and the stability of functional groups within BHR, examining these aspects from both temporal and spatial perspectives.

### 3.3. Composition and Succession Characteristics of Functional Groups

**Figure 6:**
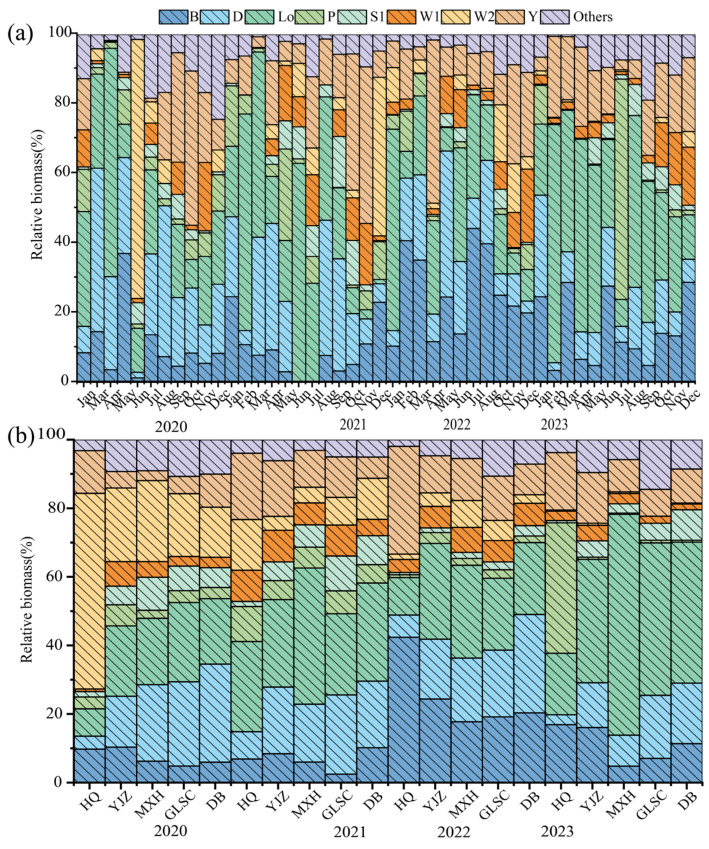
The relative biomass of the dominant phytoplankton functional groups in BHR exhibited temporal (**a**) and spatial (**b**) fluctuations.

Using the phytoplankton functional group classification put forth by Reynolds et al. [3] and Padisák et al. [5], the study area encompassed a total of 27 distinct functional groups of phytoplankton, and their habitat characteristics and representative species were shown in Table 1. Functional groups exhibiting a relative biomass exceeding 5.00% were defined as the dominant functional groups within the ecosystem [21]. During the study period, there were 8 dominant functional groups of phytoplankton in BHR (B, D, L_O_, P, S1, W1, W2, Y), and the remaining 19 functional groups were called other functional groups. The biomass of phytoplankton functional groups in BHR varied from 4.56 × 10^−3^ to 74.38 mg/L in time and from 8.19 × 10^−3^ to 64.37 mg/L in space. The spatiotemporal variation in the relative biomass of phytoplankton dominant functional groups was shown in Figure 6.

The interannual succession characteristics of phytoplankton dominant functional groups in BHR were 2020 (W2/L_0_/D/Y/B → 2021 (L_0_/D/Y/W2/W1/P/B/S1 → 2022 (B/L_0_/Y/D/W1) → 2023 (L_0_/P/Y/B/D). Five dominant functional groups were detected in 2020, 2022, and 2023, accounting for 82.67%, 88.64%, and 89.50% of the total biomass, respectively. Eight dominant functional groups were detected in 2021, accounting for 95.87% of the total biomass. The spatial succession characteristics of phytoplankton dominant functional groups were HQ (P/B/Y/L_0_/W2) → YJZ (L_0_/B/D/Y/W1) → MXH (L_0_/D/Y/B) → GLSC (L_0_/D/Y/B/S1) → DB (L_0_/D/B/Y/S1). Five dominant functional groups were detected in HQ, YJZ, GLSC, and DB, accounting for 82.29%, 88.80%, 80.32%, and 77.53% of the total biomass, respectively. Four dominant functional groups were detected in MXH, accounting for 80.52% of the total biomass.

Whether in temporal or spatial succession, the three functional groups B, L_0_, and Y were dominant functional groups. The B functional group is represented by the algae species *Cyclotella* sp.; the L_0_ functional group is represented by the algae species *Merismopedia* sp. and *Peridinium* sp.; and the Y functional group is represented by the algae species *Cryptomonas* sp. The spatiotemporal distribution differences in phytoplankton dominant functional groups in BHR were mainly reflected in the changes in biomass and the proportion of each functional group.

### 3.4. Spatiotemporal Succession Rate of Functional Groups

**Figure 7:**
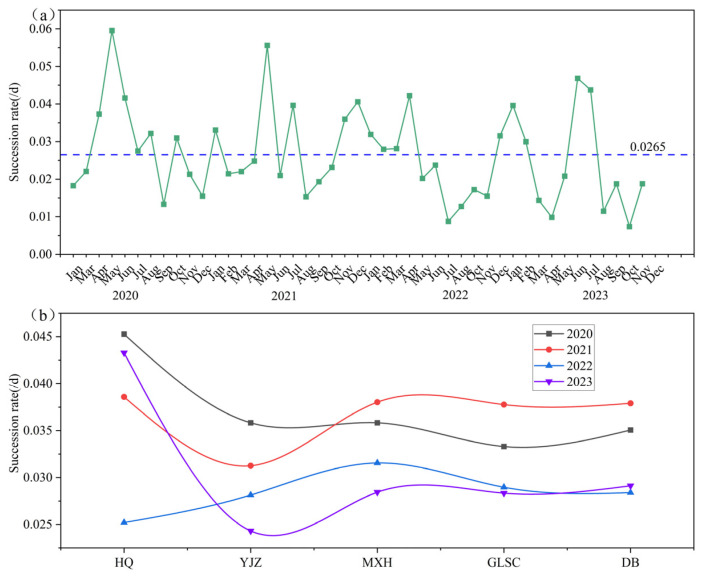
Monthly average succession rate (**a**) and spatial average succession rate (**b**) of functional groups from 2020 to 2023 (the blue dashed line denotes the mean succession rate).

The succession rate (SR) of phytoplankton can be utilized as a basis for determining the succession characteristics of phytoplankton [7]. Employing SR analysis for phytoplankton functional groups facilitates the detection of pivotal temporal shifts within the phytoplankton functional group composition in the reservoir. The monthly SR of phytoplankton functional groups from 2020 to 2023 is shown in Figure 7a (succession rate to three decimal places). The SR of phytoplankton functional groups in BHR was between 0.007 and 0.060/d, with an average of 0.027/d, and the highest SR mainly occurred from April to June. The annual SR of the phytoplankton functional groups did not show any statistically significant variation (*p* > 0.05, *n* = 45).

The spatial SR (Figure 7b) shows that except for 2022, the highest SR of the functional group occurs at HQ point, and the lowest SR occurs at YJZ. Throughout the study period, the average SR of functional groups at the five sites were 0.038 (HQ), 0.030 (YJZ), 0.033 (MXH), 0.032 (GLSC), and 0.033 (DB), respectively. In general, the average SR at HQ was the highest, and the SR at MXH, GLSC, and DB did not fluctuate.

**Figure 8:**
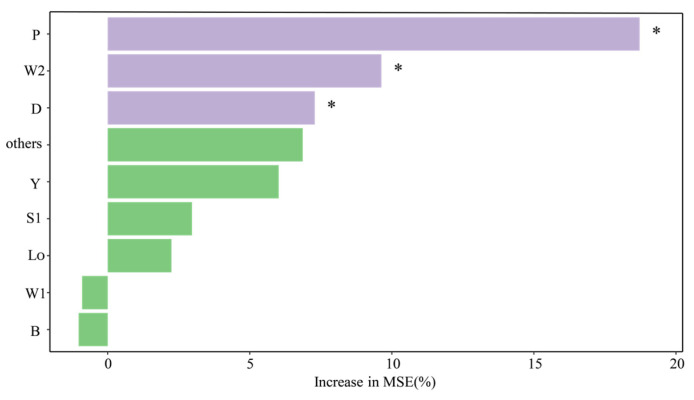
Significance analysis of Random Forest (* indicates the difference level).

Random Forest is a powerful machine learning technology that is widely used in classification and regression problems. The Random Forest model is employed to rank the importance of variables. A higher score indicates a more substantial influence on the model’s predictive outcomes [22]. The key functional groups affecting the SR were identified through Random Forest. The results showed that the P, W2 and D functional groups were strong predictors of the SR of BHR, and the P functional group had the strongest predictability (Figure 8).

The Mantel test constitutes a statistical method employed to ascertain the correlation between two datasets. These two sets of data are usually represented as distance or similarity matrices. The Mantel test assesses whether they are correlated by comparing the correlation between the two matrices [23]. With environmental variations, functional groups exhibit adaptive changes. The Mantel test analysis demonstrated significant associations between these functional groups and aquatic physicochemical parameters.

**Figure 9:**
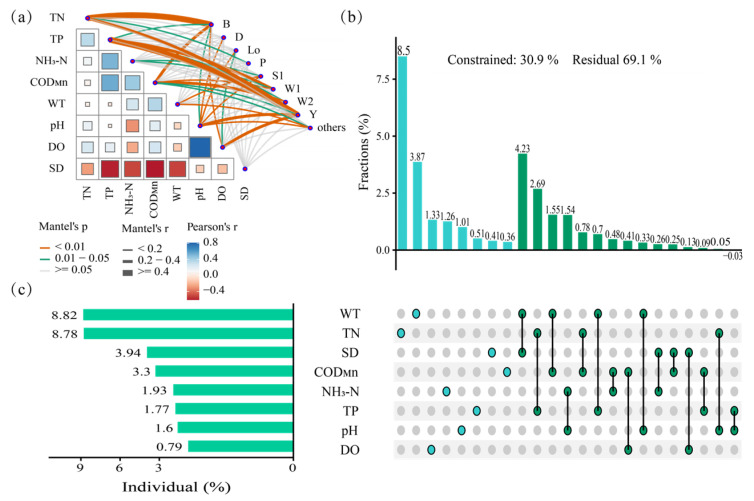
The Mantel test shows the relationship between environmental factors and functional groups (**a**); variance partitioning results were visualized through an upset matrix plot, with vertical bars representing combined explanatory power of environmental factors on phytoplankton functional groups (**b**) and horizontal bars depicting the individual contributions of environmental factors (**c**).

The upset chart is used to visualize the intersection and difference between multiple sets and is especially suitable for displaying the complex relationship between multiple sets of data [24]. Its main components include the intersection matrix and auxiliary graphics, which can clearly show the different combinations in the dataset. The graphical representation within the chart denotes the proportion of variance elucidated by the respective environmental variables. The point matrix, accompanied by the superordinate bar diagram, delineates the quantitative measures of both communal and distinctive contributions. As a result of the modification to the mean square of R, the contribution of negative values can be ignored. The negative contributions in the figure are not completely displayed, but they are incorporated in the tallying of the cumulative contribution for each variable type, and the residuals represent the unexplained parts of these variables. Hierarchical partitioning was employed to examine the correlation between the abundance of phytoplankton functional groups and environmental factors, as depicted in Figure 9. Through the visual hierarchical partitioning results of the upset diagram, it can be seen that the RDA interpretation rate of environmental factors on the abundance of functional groups was 30.9%, and the contribution rate of physical and chemical indicators from large to small was WT, TN, SD, COD_Mn_, NH_3_-N, TP, pH, and DO.

The two analytical approaches examine the relationships between environmental factors and functional groups from distinct perspectives, leading to partially divergent outcomes. Nevertheless, WT, TN, and CODMn consistently emerged as predominant drivers in phytoplankton functional group succession. By elucidating the driving mechanisms of environmental parameters on functional groups dynamics through these methodologies, we can develop rational regulation strategies to modulate the developmental trajectories of functional groups, thereby maintaining the ecological equilibrium of aquatic communities.

### 3.5. Functional Group Community Stability Analysis

The average variation degree (AVD) is an index to measure the average degree of species abundance changes in a biological community. It reflects the overall stability of the community by calculating the variation between the abundance of each species in the community at a certain time or condition and its abundance at another time or condition. The smaller the AVD, the smaller the change in species abundance in the community, that is, the species composition of the community is relatively stable [25].

**Figure 10:**
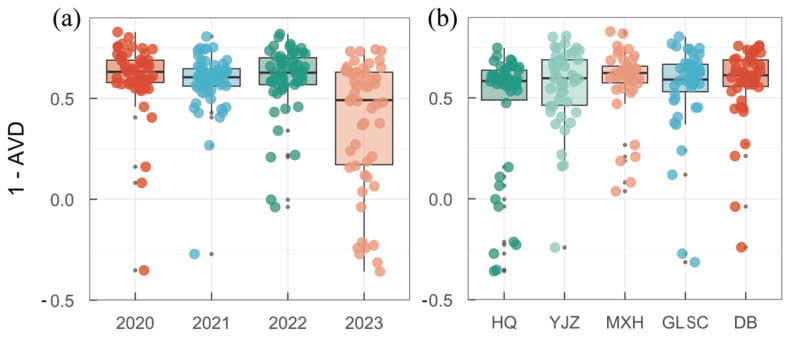
Temporal (**a**) and spatial (**b**) variations in the AVD of the phytoplankton functional group community in BHR.

In order to analyze the community stability of phytoplankton functional groups in BHR, the temporal and spatial variability of AVD in BHR was calculated (Figure 10). The larger the value of 1-AVD in the figure, the more stable the community is. The average AVD in 2020, 2021, 2022, and 2023 was 0.428, 0.417, 0.405, and 0.745, respectively. The average AVD in 2023 was the highest. The AVD analysis revealed that the stability of the functional group community structure was the lowest in 2023. The average AVD for HQ, YJZ, MXH, GLSC, and DB was 0.692, 0.442, 0.503, 0.450, and 0.426, respectively. The average AVD for HQ was the highest. The AVD analysis showed that the stability of the HQ functional group community structure was the lowest.

**Figure 11:**
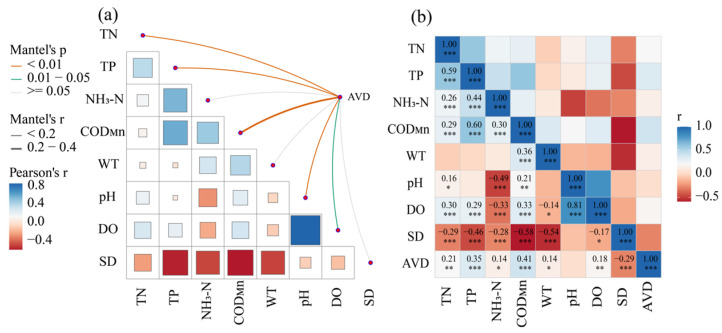
Mantel test (**a**) and heatmap (**b**) of AVD and environmental factors of BHR (*, **, *** in the figure represent the significance levels of *p* < 0.05, 0.01 and 0.001 respectively).

In order to reveal the environmental driving factors affecting the function group AVD, Mantel test analysis was conducted on the AVD and environmental factors of BHR (Figure 11a). The results indicate that TN, TP, and COD_Mn_ exhibited a highly significant positive correlation with AVD, while the DO showed a significant positive correlation with AVD, pH displayed a highly significantly negative correlation with AVD, and COD_Mn_ had the strongest correlation with AVD (Figure 11b).

**Figure 12:**
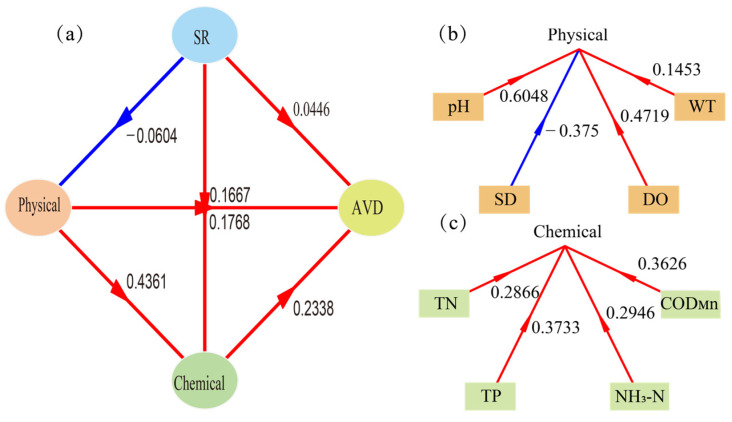
The relationship between the SR, AVD, physical factors, and chemical factors (**a**) Weight of each physical factor (**b**) Weight of each chemical factor (**c**) Red lines denote positive impacts, whereas blue lines signify negative impacts.

To ascertain the relationship between the SR, AVD, physical factors (WT, pH, DO, SD), and chemical factors (TN, TP, NH_3_-N, COD_Mn_) of the functional group of BHR, PLS-PM was applied to the four factors, which did not require high data normality and independence, and has been widely used to study the complex multivariate relationship between variables [26]. The PLS-PM analyses identified interactions between all four of the SR, AVD, physical factors and chemical factors (Figure 12a).

The color of the arrow between the SR and the AVD is red, indicating that the SR has a positive impact on the AVD, that is, the higher the SR of functional groups, the greater the community variability and the more unstable the community. The arrow between SR and physical factors is blue, indicating that the SR has a negative impact on physical factors. Physical factors have a positive impact on chemical factors, and both physical factors and chemical factors have a positive impact on the AVD. Among the physical factors, pH and DO account for the largest weight (Figure 12b), while TP and COD_Mn_ account for the largest weight in the chemical factors (Figure 12c), demonstrating that these four environmental variables played a crucial role in the model.

## 4. Discussion

### 4.1. Spatiotemporal Succession Characteristics of Functional Groups

Factors such as water chemistry, hydrological environment, and human disturbance have important effects on the spatiotemporal distribution of phytoplankton functional groups. Under different temporal and spatial heterogeneity conditions, different dominant functional groups appear, and the suitable habitat of dominant functional groups can better reflect the characteristics of water quality [27]. A total of 27 functional groups were detected during the study, including 8 dominant functional groups. There were spatiotemporal differences in the distribution of phytoplankton functional groups in BHR, but B, L_0_, and Y occupied a dominant position in both temporal and spatial succession.

The main growth temperature of the B functional group is 16–26 °C, and the water temperature of BHR is 7.10–28.30 °C. The water conditions are conducive to the swift proliferation of B functional groups, specifically *Cyclotella* sp. [28]. *Peridinium* sp. constitutes the quintessential representative algal species within functional group L_0_, exhibiting a wide range of adaptability to temperature. *Peridinium* sp. has flagella and can obtain nutrients through free movement. Therefore, *Peridinium* sp. occupies a dominant position during the entire study period [29]. Functional group Y is represented by *Cryptomonas* sp., which are suited to various environments and have a large specific surface area that allows them to rapidly absorb nutrients from the water column, while their flagella give them a competitive advantage [30]. The suitable habitats of the three dominant functional groups are mesotrophic to eutrophic conditions, indicating that the water nutrition of BHR is mesotrophic to eutrophic [31].

The water quality monitoring of BHR from July 2009 to August 2011 shows that its TN and TP exceeded the Class IV standard in the national surface water environmental quality standard [32]. During the period from October 2015 to September 2016, no significant fluctuations were detected in DO concentrations in BHR. Overall, BHR was classified as mesotrophic based solely on TP concentrations. However, TN concentrations slightly exceeded the eutrophication threshold of 1.5 mg/L. According to the *Environmental Quality Standards for Surface Water (GB 3838-2002)*, the measured TN levels align with Class IV water quality criteria [33]. The conclusions of this article are similar to those of these studies.

The Random Forest analysis indicates that the key functional groups dominating the succession rate of BHR were P, W2, and D functional groups. The P functional group gives the strongest prediction for the succession rate of functional groups. It may be that the P functional group is mainly composed of Bacillariophyta, which have a high utilization efficiency of nutrients and are suitable for growing in mesotrophic to eutrophic water bodies [34,35]. The maximum succession rate of the functional groups from 2020 to 2024 mainly occurred from April to June. There may be two reasons for this. One is that the high temperature promotes the growth of phytoplankton due to the increase in temperature from April to June [36]. Second, because the reservoir water level rose during this period, the high water level was conducive to the growth of phytoplankton, and the water agitation led to the rapid change in phytoplankton biomass [37,38].

The SR results demonstrate that the SR of the HQ functional groups was higher than that of other sites, except for in 2022. The high SR of functional groups at the HQ point may be due to the fact that HQ is located at the head of BHR, which receives nutrient input from upstream, and the water flow velocity at the head of the reservoir is fast, which is conducive to the mixing and distribution of nutrients and fosters the growth and succession of phytoplankton. Therefore, the dynamic changes in the phytoplankton Bacillariophyta abundance in BHR can better predict the succession rate of functional groups, especially at the HQ point. Diatoms also typically are less buoyant than other taxa, so higher turbulence would aid them remaining in the photic zone.

### 4.2. Main Environmental Factors Driving the Succession of Functional Groups

Various functional groups are influenced by distinct environmental factors with differing levels of impact. The environmental factors that affect the spatiotemporal distribution pattern of functional groups were divided by adopting the Mantel test and hierarchical partitioning.

The Mantel tests demonstrate significant correlations (*p* < 0.01) between phytoplankton functional groups and key parameters: functional groups B, P, W1, W2, and Others showed positive associations with TN, COD_Mn_, TP, and WT, while functional groups D, Lo, S1, and Y exhibited negative correlations with pH, with functional group Y additionally correlating positively with TP and DO.

The importance ranking results indicated that WT, TN, and SD were the top three factors affecting the succession of functional groups. The total interpretation rate of environmental factors on the spatiotemporal succession of functional groups was 30.90%, and the interpretation rates of WT, TN, and SD were 8.82%, 8.78%, and 3.94%, respectively, accounting for 69.71% of the total interpretation rate. The single interpretation rate of WT for functional group succession was 3.87%, the single interpretation rate of TN for functional group succession was 8.5%, the single interpretation rate of SD for functional group succession was 0.41%, and the joint interpretation rate of WT and SD for functional group succession was 4.23%.

A substantial body of research has demonstrated that temperature and nutrient levels were the most critical determinants of phytoplankton dynamics [39,40]. A study on the dynamic driving factors of phytoplankton in Chaohu Lake and Honghu Lake also further validates the results of the present study [41,42].

The growth of algae is influenced by WT, which impacts the speed of enzymatic reactions involved in their photosynthesis and respiration processes. The increase in WT will affect the photosynthesis and respiration of phytoplankton and then directly or indirectly affects the change in community structure [43]. At the appropriate temperature, an increase in water temperature can promote the rapid growth of algae [44]. The growth of phytoplankton is dependent on appropriate temperature conditions. For example, Cyanophyta and Chlorophyta are suitable to grow in high-temperature environments, whereas Bacillariophyta can grow rapidly in low temperature environments [45,46].

Nitrogen nutrients are necessary for algae growth. An increase in nitrogen nutrient concentration in water provides favorable conditions for the growth of phytoplankton [47]. Previous research has indicated that a representative species of the B functional group, *Cyclotella* sp., has a high demand for nitrogen and an efficient utilization ability, and more easily forms a dominant population in water with high TN concentration [28].

SD is a physical factor that can directly reflect the water quality. Generally, an increase in phytoplankton concentration corresponds to a decrease in water body transparency. A high SD will decrease the light transmittance of water and directly affect the light utilization rate of phytoplankton; even if the temperature and nutrients reach the optimal growth conditions, phytoplankton cannot grow rapidly, and the impact on phytoplankton in the reservoir will increase with the increase in the nutritional status of the reservoir [48]. Overall, WT, TN, and SD are the primary environmental variables influencing the succession of phytoplankton functional groups within BHR.

### 4.3. Stability Characteristics and Driving Factors of Functional Groups

The results of the AVD analysis reveal that there were spatiotemporal differences in the AVD of phytoplankton functional groups in BHR, and the AVD in 2023 was higher than that in other years. The spatial AVD analysis results were similar to the SR of the functional groups, and the AVD of HQ was higher than that of the other four points.

The Mantel test indicates that TN, TP, COD_Mn_, and DO had a significant positive correlation with AVD, while pH was significantly negatively correlated with AVD. The correlation between COD_Mn_ and AVD was the most significant, showing a very significant positive correlation. COD_Mn_ serves as a crucial parameter for indicating the concentration of organic matter in water. An increase in COD_Mn_ values corresponds to a higher level of water pollution due to a variety of organic compounds and other reducing agents. Organic matter is the nutrient source of phytoplankton growth and the key factor affecting phytoplankton growth [49]. Low COD_Mn_ will inhibit the growth of phytoplankton, and the content of organic matter will affect the composition of the phytoplankton community and then affect the stability of the community [50].

In 2023, COD_Mn_ was significantly higher than that in 2020, 2021, and 2022 (Figure 2), so the AVD of BHR in 2023 was also higher than that in other years. The phenomenon of eutrophication in the HQ section of BHR is affected by the inflow of polluted water from the tributary Dongmenqiao River on the one hand and is also due to the large number of residential areas, high intensity of human activities, and large base of domestic sewage in the HQ section, but its water area is small, and its self-purification capacity is limited.

The PLS-PM showed that the SR not only directly affected AVD but also indirectly affected the chemical factors and AVD by affecting the physical factors. Physical and chemical factors had a positive effect on each other and had the highest weights, suggesting that physical and chemical factors are interrelated and interact in ecosystems. The community AVD was influenced by several factors, including SR and physical and chemical factors, reflecting the complexity of community variability in ecosystems. Therefore, the positive correlation between COD_Mn_ and AVD may also be the result of a variety of environmental and biological factors.

### 4.4. Implications for Ecosystem Management of Baihua Reservoir

Based on the relationship among the succession rate of phytoplankton functional groups, community stability, and environmental factors, several insights can be proposed for the management of the Baihua Reservoir’s aquatic ecosystem.

Firstly, it is essential to regularly monitor factors related to the succession rate of phytoplankton, such as P functional groups, WT, TN, TP, and SD. This monitoring will help to timely assess the health status of the ecosystem and predict potential ecological changes. Secondly, controlling the nutrient input is crucial. Since the TN, TP, and COD_Mn_ directly influence the growth of phytoplankton and community stability, management measures should focus on regulating nutrient discharges from agricultural and industrial activities to mitigate the risk of eutrophication in water bodies. Furthermore, maintaining water transparency is vital, as the SD affects the efficiency of photosynthesis, thereby influencing phytoplankton growth. Thus, management strategies should include the cleaning and protection of water bodies to reduce the input of suspended solids, thereby enhancing the water transparency and promoting the healthy growth of phytoplankton. Lastly, the establishment of an algal warning system is recommended. This system should be based on monitoring data to create a warning mechanism for the succession rate of phytoplankton and community stability. In the event of any anomalous changes, timely interventions should be implemented.

By adopting these measures, the health of the aquatic ecosystem can be significantly enhanced, promoting the stable growth of phytoplankton and achieving sustainable management of the ecosystem.

## 5. Conclusions

The phytoplankton in BHR can be divided into 27 functional groups, including 8 dominant functional groups, namely B, D, L_0_, P, S1, W1, W2, and Y. There were differences in the spatiotemporal succession characteristics of functional groups, but B, L_0_, and Y occupied dominant positions in the temporal and spatial succession, indicating that the water nutrition status of BHR was in a mesotrophic to eutrophication state. The AVD in 2023 was higher than that in other years, indicating that the average variability of the functional group community of BHR in 2023 was high. The SR and AVD of the HQ site were higher than at the other four sites, which proved that the community structure of the HQ site functional group was updated rapidly. SR not only has a direct positive relationship with AVD but also indirectly affects AVD by affecting physical and chemical factors. These findings provide enlightenment for the management of the water ecosystem of Baihua Reservoir.

## Figures and Tables

**Table 1:** Habitat characteristics and representative species of the phytoplankton functional groups in Baihua Reservoir.

Functional Group	Habitat Characteristics	Representative Species	Tolerance	Susceptibility
A	Oligotrophic, clean, deep water	*Rhizosolenia*	Low nutrients	Elevated pH
B *	Mesotrophic trophic, small-to-medium, or large shallow-water bodies	*Cyclotella* sp.	Low light	Elevated pH, water stratification
C	Eutrophic, small- and medium-sized reservoirs	*Asterionella* sp.	Low light	Water stratification
D *	Rich in nutrients, cloudy	*Synedra* sp., *Nitzschia* sp.	Scouring	Nutrient deficiency
E	Oligotrophic or heterotrophic type, small water bodies, shallow water	*Dinobryon divergens*	Low nutrients	CO_2_ deficiency
F	Mesotrophic-to-eutrophic, clean, and strong water mixing	*Oocystis* sp., *Kirchneriella* sp.	Low nutrients	CO_2_ deficiency
G	Eutrophic, stagnant water bodies	*Eudorina* sp., *Pandorina* sp	High light	Nutrient salt deficiency
H1	Eutrophic, stratified, low nitrogen content	*A nabaena* sp., *Aphanizomenon* sp.	Low carbon/nitrogen content	Water mixing, low light, low phosphorus
J	High nutrient, mixed, shallow water	*Scenedesmus* sp., *Crucigenia* sp., *Tetraedron* sp.		High light
K	Eutrophic, shallow water	*Aphanocapsa* sp.		Strong water mixing
L_M_	eutrophic to hypereutrophic, small- and medium-sized water bodies	Dactylococcopsis sp., *Ceratium* sp.	Extremely low carbon content	Water mixing, low light
L_O_ *	Mesotrophic-to-eutrophic, medium-to-large water bodies, can be deep or shallow	*Merismopedia* sp., *Chroococcus* sp., *Peridinium* sp.	Nutrient stratification	Prolonged/deep mixing
MP	Frequent agitation, turbidity, shallow water	*Oscillatoria* sp., *Navicula* sp., *Achnanthes* sp.	Mixing disturbance	
N	Continuous or semi-continuous mixed bodies of water	*Cosmarium* sp.	Low nutrients	Water stratification, elevated pH
P *	It is similar to functional group N, but the trophic status of the water body is higher	*Melosira* sp., *Fragilaria* sp., *Closterium* sp.	Low light, low carbon content	Water stratification, silicon deficiency
S1 *	The mixture is cloudy and has low transparency	*Pseudanabaena limnetica*, *Rhabdogloea*, *Limnothrix* sp.	Extremely low light	Scouring
S2	Warm, highly alkaline, shallow water	*Spirulina*	Low light	Scouring
S_N_	Warm, blended	*Cylindrospermum raciborskii*, *Raphidiopsis*	Low light, low nutrients	Scouring
T	Mix water bodies continuously	*Mougeotia, Quadrigula chodatii*	Low light	Nutrient deficiency
TC	Eutrophication, still or flowing water, with upright plants	*Lyngbya* sp.		Scouring
W1 *	Organic pollution, shallow water	*Euglena* sp. *Euglena oxyuris*	High biochemical oxygen demand (BOD)	Grazing pressure
W2 *	Mesotrophic nutrition, shallow water	*Trachelomonas* sp.		
X1	Super nutritious, shallow water	*Chlorella* sp., *Ankistrodesmus* sp.	Water stratification	Nutrient deficiency, filter feeding
X2	Mesotrophic-to-eutrophic, shallow water	*Chlamydomonas* sp.	Water stratification	Water mixing, filter feeding
X3	Oligotrophic, mixed, shallow water	*Schroederia* sp.	Harsh environmental conditions	Water mixing, grazing pressure
Y *	Still-water environment	*Cryptomonas* sp. *Gymnodinium* sp.	Low light	Predation
Z	Oligotrophic	*Synechococcus*	Low nutrients	Low light, grazing pressure

* Indicates the dominant functional group.

## Data Availability

All data were included in the paper.
